# Effects of Chicken Skin Protein Hydrolysate and Bone Protein–Mineral Mass on the Quality of Emulsified Poultry Sausages

**DOI:** 10.3390/foods15061091

**Published:** 2026-03-20

**Authors:** Anuarbek Suychinov, Eleonora Okuskhanova, Zhanibek Yessimbekov, Aitbek Kakimov, Guldana Kapasheva, Baktybala Kabdylzhar, Rasul Turagulov

**Affiliations:** 1 Kazakh Research Institute of Processing and Food Industry (Semey Branch), Semey 071410, Kazakhstan; asuychinov@gmail.com (A.S.); gena.89.89@mail.ru (G.K.); baktybala.20@mail.ru (B.K.); 2 Research School of Food Engineering, Shakarim University, Semey 071412, Kazakhstan; eokuskhanova@gmail.com (E.O.); bibi.53@mail.ru (A.K.); rassul007@mail.ru (R.T.)

**Keywords:** poultry by-products, protein hydrolysate, protein–mineral mass, emulsified sausages, functional properties, waste valorization, sustainable meat processing

## Abstract

The poultry industry generates large amounts of protein- and mineral-rich by-products that remain underutilized. This study investigated the use of chicken skin protein hydrolysate and chicken bone protein–mineral mass (PMM) as functional ingredients in emulsified poultry sausages. The hydrolysate was characterized by a high protein content (52.25%) and high water- and fat-binding capacity (142% and 125%, respectively), while the PMM served as a source of protein and minerals with stable physicochemical and rheological characteristics. These ingredients were incorporated into sausage formulations at different substitution levels. Partial replacement of poultry meat increased protein and mineral content and affected key technological properties, including water-binding capacity, emulsion stability, cooking loss, and shear force. Moderate inclusion levels were associated with a more cohesive protein matrix, lower cooking losses, and improved structural stability, whereas excessive substitution resulted in increased firmness and less favorable sensory characteristics. Among the tested formulations, the combination of 18% PMM and 4% protein hydrolysate showed the most balanced technological and sensory performance. The findings suggest that poultry by-products processed into functional ingredients may have potential for application in value-added sausage formulations.

## 1. Introduction

The poultry industry is one of the fastest-growing sectors of global animal protein production, driven by population growth, urbanization, and increasing demand for affordable, nutritionally balanced foods. Poultry meat is widely recognized for its high biological value, favorable amino acid composition, and relatively low production cost compared with red meats [1]. Globally, the continuous expansion of poultry processing capacity has been accompanied by a proportional increase in secondary raw materials and by-products generated during slaughtering and further processing [2,3].

In the Republic of Kazakhstan, poultry production has shown steady growth in recent years. Poultry meat output reached approximately 360,000 tonnes in 2024, while production in the first ten months of 2025 exceeded 307,000 tonnes, reflecting continued expansion of the sector [4]. Between 2021 and 2024, production increased by about 27%, and domestic self-sufficiency rose from 58% to nearly 79%, highlighting the growing importance of poultry production for national food security [5]. At the same time, intensified poultry processing generates substantial quantities of by-products, including skin, bones, feet, heads, necks, and feathers. These secondary raw materials may account for 30–45% of live poultry weight and are often underutilized or directed to low-value applications [6]. Their inefficient use leads to economic losses and increases environmental pressure through waste accumulation and associated pollution risks [7].

In response to these challenges, both industry and the scientific community are actively developing advanced processing technologies aimed at the valorization of poultry by-products. Among these approaches, enzymatic and thermo-enzymatic hydrolysis have gained particular attention due to their ability to convert collagen-rich raw materials into high-value protein hydrolysates and protein–mineral products. Enzymatic hydrolysis, when conducted under controlled conditions, allows selective cleavage of peptide bonds while preserving nutritional quality and functional properties of the resulting peptides [8,9]. Compared with chemical hydrolysis, enzymatic methods are more environmentally friendly, generate fewer undesirable by-products, and enable precise control of the degree of hydrolysis.

Despite the growing body of research on the enzymatic and microbial processing of poultry by-products, most published studies have focused on the production, optimization, and characterization of isolated hydrolysates or mineral-rich fractions rather than their direct use in finished meat products [10,11,12,13,14]. Previous work has demonstrated the feasibility of obtaining poultry-derived hydrolysates with high protein content, favorable amino acid composition, improved digestibility, low-molecular-weight peptides, and, in some cases, antioxidant activity or feed-related functionality [15,16,17,18,19]. However, their combined application in meat systems remains insufficiently investigated. In particular, there is limited information on how chicken skin protein hydrolysate and chicken bone protein–mineral mass interact within emulsified sausage matrices and how their simultaneous incorporation affects composition, water binding, emulsion stability, texture, cooking loss, microstructure, color, and sensory quality. Therefore, an important knowledge gap remains regarding the optimal formulation level at which these poultry-derived ingredients can improve product quality without causing excessive firmness, color deterioration, or sensory defects.

The novelty of the present study lies in the combined use of two functional ingredients obtained from different poultry by-products (chicken skin protein hydrolysate and chicken bone protein–mineral mass) in emulsified poultry sausages. The present work evaluates their integrated technological performance in a finished meat product and identifies the formulation range that provides the best balance between nutritional enrichment, processing stability, and consumer acceptability.

Accordingly, this study aimed to evaluate the effects of chicken skin protein hydrolysate and chicken bone protein–mineral mass on the physicochemical, functional–technological, structural, and sensory properties of emulsified poultry sausages and to determine their optimal inclusion levels. It was hypothesized that moderate incorporation of these ingredients would increase protein and mineral content, improve water retention and structural stability, and maintain acceptable sensory quality.

## 2. Materials and Methods

### 2.1. Samples

Chicken skin and bones were obtained from a commercial poultry processing facility, Vostok-Broiler LLC (Semey, Kazakhstan), immediately after poultry slaughter. The raw materials were transported to the laboratory in insulated portable coolers with the temperature maintained at (+4)–(+6) °C. To ensure uniform composition, raw materials were collected from the same processing batch. Upon arrival at the laboratory, the raw materials were frozen and stored at −18 °C for 24 h before subsequent experimental processing and analysis. The workflow of the study, including preparation of protein hydrolysate and protein–mineral mass, sausage production, and analytical procedures, is illustrated in Figure S1 (Supplementary File).

### 2.2. Preparation of Chicken Skin Protein Hydrolysate

Chilled chicken skin obtained during the primary processing of poultry meat was used as a collagen-containing raw material. Before enzymatic treatment, the skin was mechanically comminuted without the addition of water. Grinding was carried out in two consecutive stages using a colloid mill. The initial temperature of the raw material before grinding was 4 °C. After the first grinding stage, the temperature increased to 13.5 °C, and after the second stage to 17.6 °C. These values did not exceed the critical threshold for native collagen and therefore did not induce thermal denaturation, ensuring conditions suitable for controlled enzymatic hydrolysis.

Enzymatic hydrolysis of the ground chicken skin was carried out using a two-stage process involving proteolytic enzymes of different origins. In the first stage, the enzyme preparation Enzimix-U (Endocrine Enzyme Plant LLC, Moscow, Russia) was applied at a dosage of 0.2% (*w*/*w*) relative to the mass of collagen-containing raw material (equivalent to 2 g·kg^−1^).

The enzyme was dissolved in cheese whey at 35–36 °C, after which the solution was mixed with the minced chicken skin at a raw material–liquid ratio of 1:1.5. Hydrolysis was conducted at 40 °C and pH 5.5 for 3 h under thermostatically controlled conditions. These parameters were selected to ensure controlled proteolysis and partial disintegration of connective tissue structures. Process progress was monitored through controlled reaction time and technological parameters (temperature, pH, and mixing), which were selected based on preliminary optimization experiments and previously reported processing conditions. After completion of the first hydrolysis stage, the liquid phase was separated and the solid fraction was rinsed with potable water to remove soluble hydrolysis products and residual enzyme.

In the second stage, intensive hydrolysis was conducted using “Protozyme S” (Biopreparat LLC, Moscow, Russia), an alkaline fungal protease with a declared activity of 50,000 U/g. The enzyme exhibits activity over a wide pH range (5.5–11.5) with optimal conditions at pH 8.0–10.5 and 50–60 °C. The enzyme was added at a dosage of 10% relative to the mass of raw material, and hydrolysis was carried out at 55 °C for 6 h.

After completion of hydrolysis, the enzymatic reaction was terminated by thermal inactivation. The hydrolysate was heated to 90 ± 2 °C for 10 min, which ensured complete denaturation and inactivation of the proteolytic enzymes and stabilization of the hydrolysis products.

After completion of enzymatic hydrolysis and thermal inactivation of the enzyme, the reaction mixture was separated into liquid and insoluble fractions. The supernatant containing soluble peptides and proteins was collected and used as the hydrolysate fraction, while the insoluble residue was discarded. The collected supernatant was subsequently subjected to freeze-drying at −55 °C under a residual pressure of 10 kPa for 18–24 h, until the moisture content did not exceed 7%, yielding a stable powdered protein hydrolysate.

### 2.3. Preparation of Chicken Bone Protein–Mineral Mass

Chicken poultry by-products, specifically wings and necks, were used as raw materials to produce the protein–mineral mass via enzymatic bioconversion with pepsin. The raw materials were washed under running water and initially minced using a meat grinder equipped with a 3 mm perforated plate, followed by further comminution in a colloid mill to achieve an average particle size of approximately 0.1 mm.

To prepare the suspensions, different water-to-raw-material ratios (hydromodules) were applied depending on the type of raw material: 1:4 (*w*/*w*) for bone-containing material and 1:3 (*w*/*w*) for necks, accounting for differences in mineral content and tissue density. The suspensions were acidified with 1 M HCl to a target pH of 2.0 ± 0.1 and thermostatted at 40 ± 2 °C.

Pepsin was used as the hydrolyzing enzyme, with dosage calculated based on the protein content of the raw materials. The enzymatic activity applied in the experiments was 1000 U·g^−1^ protein, corresponding to approximately 0.50 g pepsin for bone material and 0.75 g pepsin for neck material (enzyme activity 100,000 U·g^−1^). The enzyme was pre-dissolved in 20–30 mL of acidified water (pH 2–3) and added to the suspension under continuous mixing.

Enzymatic hydrolysis was carried out at pH 2.0–2.2 and 40 ± 2 °C for 2.5–3.0 h for bone material and 2.0–2.5 h for necks, with stirring every 15 min and pH monitoring every 30 min. The reaction was terminated by neutralization to pH 7.0–7.2 using 1 M NaOH, followed by heat treatment at 85–90 °C for 5–10 min to inactivate the enzyme. The resulting mass was centrifuged at 3000–5000 rpm for 15–20 min to remove excess liquid, yielding a homogeneous minced paste defined as the protein–mineral mass.

Enzymatic hydrolysis was carried out at pH 2.0–2.2 and 40 ± 2 °C for 2.5–3.0 h for bone material and 2.0–2.5 h for necks, with periodic mixing every 15 min and pH monitoring every 30 min. After completion of hydrolysis, the enzymatic reaction was terminated by neutralizing the medium to pH 7.0–7.2 with 1 M NaOH, followed by thermal treatment at 85–90 °C for 5–10 min to ensure complete inactivation of pepsin and stabilization of the hydrolysis products. The resulting suspension was centrifuged at 3000–5000 rpm for 15–20 min to remove excess liquid, yielding a homogeneous paste defined as the protein–mineral mass.

### 2.4. Formulation and Production of Sausage

At the next stage of the study, experimental sausage products were manufactured using protein–mineral mass (PMM) and freeze-dried chicken skin protein hydrolysate. Four formulation variants were designed to evaluate the influence of different inclusion levels of these ingredients on the technological, physicochemical, and sensory characteristics of sausages.

Variant 1 served as the control and represented a conventional frankfurter formulation containing poultry meat, starch, and standard curing ingredients without the addition of PMM or protein hydrolysate. In variants 2–4, poultry meat was partially replaced with increasing levels of PMM (15–20%) and protein hydrolysate (2–7%). In these formulations, starch was deliberately excluded to evaluate the structuring and water-binding potential of collagen-derived ingredients without the influence of an additional carbohydrate stabilizer. Consequently, the experimental design reflects both partial replacement of meat and removal of starch, allowing assessment of the ability of PMM and protein hydrolysate to function as alternative structuring components in the sausage matrix. The formulations are presented in Table 1.

### 2.5. Preparation of Sausage Mince

Poultry meat (chicken breast fillet) was minced through a 3–5 mm plate and cooled to 0–4 °C before processing. Protein–mineral mass was used in paste form, while the protein hydrolysate was applied in dry, freeze-dried form. Dry ingredients (protein hydrolysate, starch for the control variant, and spice mixture) were pre-sifted to ensure uniform distribution. The curing mixture and table salt were dissolved in 10% of the total water content.

Mincing and emulsification were performed in a bowl cutter under controlled temperature conditions. Poultry meat and protein–mineral mass were first loaded into the cutter and mixed for 2–3 min at a product temperature not exceeding 8 °C to initiate the extraction of salt-soluble proteins. Subsequently, the curing mixture and dissolved salt were added. Crushed ice or ice-cold water, protein hydrolysate, starch (control variant only), and spices were then incorporated, and chopping continued for an additional 5–6 min until a homogeneous, stable emulsion was obtained. The temperature of the sausage mince after mixing did not exceed 12 °C.

The prepared sausage mince was stuffed into casings with a diameter of 24 mm to form links 10–12 cm in length and was then subjected to a settling period at 0–2 °C for 20 min to stabilize the structure before thermal processing. Heat treatment was carried out in a multistage mode consisting of initial drying/frying at 50 °C for 30 min, followed by cooking at 68 °C for 30 min, and final heating at 78–80 °C until the internal temperature of the sausages reached 72 °C. Upon completion of heat treatment, the sausages were cooled by showering with cold water until the surface temperature decreased to approximately 25 °C and subsequently stored at 0–4 °C for 18 h before further physicochemical, structural, and sensory analyses.

### 2.6. Determination of Proximate Chemical Composition

The proximate chemical composition of the samples was determined using standardized methods in accordance with relevant GOST regulations. Protein content was quantified by the Kjeldahl method following GOST 25011-2017 [20], based on acid mineralization of organic matter with subsequent determination of total nitrogen and recalculation to protein using the prescribed conversion factor. Fat content was measured by continuous solvent extraction according to GOST 23042-2015 [21], using petroleum ether as the extractant, followed by removal of the solvent and gravimetric determination of the extracted lipid fraction. Moisture content was determined by drying the samples mixed with sand at a temperature of (103 ± 2) °C until constant mass was achieved, in accordance with standard gravimetric practice. Ash content was assessed following GOST 31727-2012 [22] by preliminary drying and charring of the samples, followed by incineration in a muffle furnace at 550 °C to constant weight, with the mineral residue calculated gravimetrically.

### 2.7. Determination of pH

The active acidity of the samples was determined in accordance with ST RK ISO 2917-2009 [23]. A water extract was prepared by homogenizing the sample with distilled water at a ratio of 1:10 (*w*/*v*). The pH value was measured at 20 °C using a calibrated portable pH meter (Seven2Go, Mettler Toledo, Greifensee, Switzerland) after prior calibration with standard buffer solutions.

### 2.8. Determination of Water Activity

Water activity (a_w_) was measured using an aWLife analyzer (Steroglass S.r.l., Perugia, Italy) in accordance with the manufacturer’s instructions. Before analysis, the instrument was calibrated with standard reference solutions to ensure measurement accuracy. Prepared samples were placed in the measuring chamber and equilibrated at 25 °C, after which the a_w_ value was automatically recorded once equilibrium conditions were achieved. Regular calibration and instrument optimization were performed to maintain the reliability and reproducibility of the measurements.

### 2.9. Determination of Water-Holding and Fat-Retention Capacities

Water-holding capacity (WHC) was determined according to a previously described method [24] using a gyrometer technique. Briefly, a 4–6 g minced meat sample was thermally treated in a sealed gyrometer at boiling temperature, and the amount of released moisture was quantified from the gyrometer scale. WHC (%) was calculated based on the difference between total moisture content and water-release capacity.

Fat-holding capacity (FHC) was determined according to a previously described refractometric method [24]. Briefly, the fat content of samples before and after thermal treatment was quantified using α-monobromonaphthalene as a solvent and refractive index measurements, and FHC (%) was calculated as the ratio of retained fat after heating to the initial fat content. All measurements were performed in replicate, and mean values were used for analysis.

### 2.10. Determination of Emulsifying Capacity and Emulsion Stability

The emulsifying capacity of the samples was evaluated using a gravimetric–centrifugation method [25]. A weighed portion of the sample (7 g) was first dispersed in 100 mL of distilled water by homogenization for 60 s at a controlled shear rate. Refined sunflower oil (100 mL) was then gradually introduced, and the mixture was further homogenized at an increased shear rate for 5 min to form a stable emulsion. The resulting emulsion was portioned into calibrated centrifuge tubes and subjected to centrifugation for 10 min under controlled conditions. After phase separation, the volume of the emulsified oil layer was measured, and the emulsifying capacity was calculated as the percentage of emulsified oil relative to the total oil added (Formula (1)).

(1) $$EC=\frac{V_{1}\cdot 100}{V}$$

where

*V*_1_—volume of emulsified oil (mL);

*V*—total volume of added oil (mL).

Emulsion stability was determined using a thermal centrifugation method [25]. Prepared emulsions were subjected to heat treatment at 80 °C for 30 min, followed by rapid cooling in water for 15 min to simulate thermal processing conditions. The treated emulsions were then distributed into calibrated centrifuge tubes and centrifuged for 5 min under controlled conditions. After centrifugation, the volume of the remaining emulsified phase was measured, and emulsion stability was evaluated based on the proportion of the stable emulsified layer relative to the total emulsion volume (Formula (2)).

(2) $$ES=\frac{V_{1}\cdot 100}{V_{2}}$$

where

*V*_1_—volume of emulsified oil after centrifugation (mL);

*V*_2_—total volume of emulsion (mL).

### 2.11. Microstructural Analysis

Microstructural observations of the meat–bone paste were performed using a low-vacuum scanning electron microscope (JSM-6390LV, JEOL, Tokyo, Japan). Samples were examined at an accelerating voltage of 15 kV, and micrographs were recorded at a magnification of ×100 [26].

### 2.12. Determination of Emulsion Viscosity

The dynamic viscosity and shear stress of the emulsions were determined using a rotational digital viscometer (BOYN, Hangzhou, China) in accordance with the manufacturer’s recommendations. An aliquot of the sample (100 mL) was placed in a thermostated 250 mL beaker and equilibrated to 18–22 °C, with temperature control ensured using a precision thermometer (±0.2 °C). The measuring spindle was immersed in the sample to the designated reference level, and measurements were carried out at a constant rotation speed of 6 rpm. Viscosity and shear stress values were recorded after 60 s of steady operation, when the instrument readings stabilized.

### 2.13. Determination of Yield Stress and Shear Force of Sausage Variants

Yield stress and shear force were determined using the universal texture analyzer Structurometer ST-2 (Radius Company, St. Petersburg, Russia) by the method described in [27]. The shear force of the sausage samples was measured to evaluate the mechanical resistance and texture of the product. Samples were prepared either using a rotating tubular knife to obtain cylindrical specimens with a diameter of 10 mm or manually cut into cubes measuring 20 mm × 20 mm. Each specimen was placed on the flat platform of a structurometer, and a cutting blade was applied to shear the sample. The force required to cut through the sample was recorded directly from the instrument. The maximum force value obtained during the test was taken as the shear force and expressed in newtons (N). Measurements were performed for each sample in triplicate, and the results were reported as mean ± standard deviation.

### 2.14. Determination of Color Characteristics

Color characteristics of the samples were determined using a portable chromameter (Chroma Meter CR-400, Konica Minolta, Japan) in accordance with standard colorimetric procedures. Measurements were carried out on the sample surface under controlled conditions, and color parameters were recorded in the CIE L*a*b* color space.

### 2.15. Sensory Evaluation

Sensory evaluation of sausage samples was carried out in accordance with [28]. The assessment was performed by a trained panel consisting of 11 assessors experienced in meat product evaluation. Prior to the study, panelists were familiarized with the evaluation procedure, scoring scale, and attribute definitions according to the referenced standard. All evaluations were conducted in a dedicated sensory laboratory under controlled conditions (air temperature 20 ± 2 °C, relative humidity 70 ± 5%, neutral lighting, and absence of extraneous odors). Samples were coded with random three-digit numbers and presented in randomized order to minimize order bias. Each assessor independently evaluated all samples without discussion. The following attributes were assessed: appearance (including cross-section), aroma, consistency (tenderness/firmness), taste, juiciness, and overall acceptability. Scoring was performed using a 5-point scale (5—excellent, 4—good, 3—satisfactory, 2—poor, 1—unacceptable), with fractional scores permitted to increase sensitivity. Each sausage variant was evaluated by all panelists, and mean values and standard deviations were calculated from individual assessor scores. The resulting data were used for statistical comparison of the sausage formulations.

### 2.16. Statistical Analysis

Each sausage formulation was produced in three independent batches (biological replicates), with each batch having a total mass of 2 kg, corresponding to approximately 40 sausages (50 g each) per batch. From each batch, five sausages were randomly selected as subsamples for physicochemical, functional, rheological, and color analyses. These sausages represented subsamples within each batch. For every subsample, analytical determinations were performed in technical triplicate, and the average value was used for statistical analysis. Results are expressed as mean ± standard deviation (SD). Statistical analysis was performed using Microsoft Excel 2016 (Microsoft Corporation, Redmond, WA, USA), Statistica 12 PL (StatSoft Inc., Tulsa, OK, USA), and SPSS IBM SPSS Statistics 29.0 (IBM Corp., Armonk, NY, USA). Differences among sausage formulations were assessed using one-way analysis of variance (ANOVA) followed by Tukey’s HSD post hoc test for pairwise comparisons. Differences were considered statistically significant at *p* ≤ 0.05.

## 3. Results

### 3.1. Physicochemical Properties of the Protein–Mineral Mass

The physicochemical, functional, rheological, and color characteristics of the protein–mineral mass (PMM) obtained from chicken bones are presented in Table 2. The PMM contained 32.61% protein, 18.65% ash, 7.79% fat, and 40.95% moisture. The measured pH was 6.93, and water activity (aw) reached 0.9701. Functional properties showed a water-binding capacity of 45.9%, water-holding capacity of 40.9%, and fat-holding capacity of 41.1%. The emulsifying capacity and emulsion stability were 48% and 18%, respectively. Rheological measurements indicated a dynamic viscosity of 7.77 Pa·s, shear stress of 5556.9 mPa, and yield stress of 0.612 kPa. Colorimetric analysis in the CIE Lab system* showed values of *L* = 48.11*, *a* = 26.11*, and *b* = 11.66*, indicating a relatively dark and reddish color of the PMM.

### 3.2. Physicochemical Properties of Chicken Skin Protein Hydrolysate

**Figure 1:**
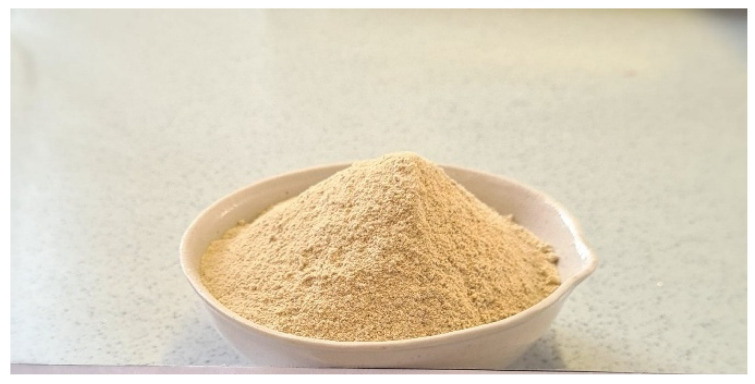
Protein Hydrolysate from chicken by-product.

The physicochemical, functional, and organoleptic characteristics of the chicken skin protein hydrolysate are presented in Table 3. The obtained hydrolysate had the appearance of a uniform fine powder with a light beige color and no detectable odor (Figure 1). Chemical composition analysis showed that the hydrolysate contained 52.25% protein, 36.79% ash, 4.82% fat, and 6.14% moisture. The measured pH was 6.19, and the water activity (a_w_) was 0.3452. Functional evaluation demonstrated a water-holding capacity of 142% and a fat-holding capacity of 125%.

### 3.3. Chemical Composition of Sausage Samples

**Figure 2:**
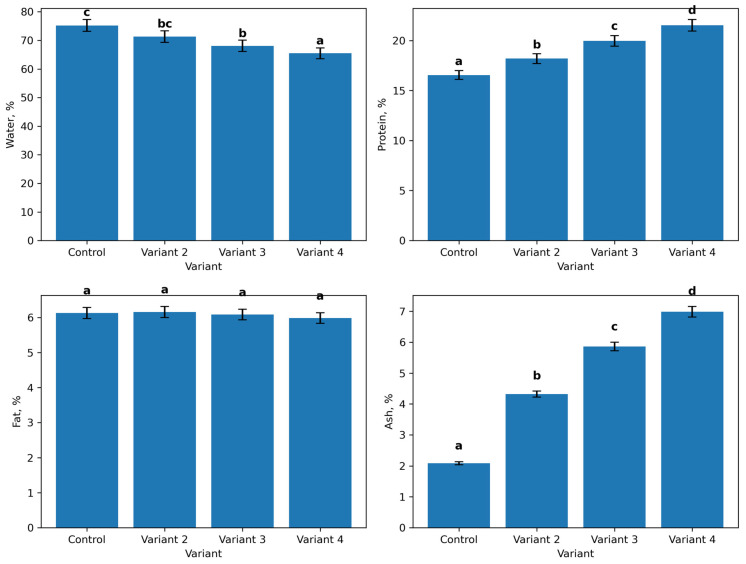
Chemical composition of different sausage mince variants. Different lowercase letters indicate statistically significant differences within each parameter (*p* < 0.05).

The chemical composition of sausage mince prepared with different levels of PMM and protein hydrolysate is presented in Figure 2. The control sample contained 75.23% moisture, 16.55% protein, 6.13% fat, and 2.09% ash. In the experimental formulations, moisture content decreased progressively from 71.33% in Variant 2 to 65.49% in Variant 4. At the same time, protein content increased from 18.19% in Variant 2 to 21.53% in Variant 4. These differences were statistically significant compared with the control (*p* < 0.05). Fat content remained relatively stable across all samples, varying within a narrow range from 5.99% to 6.16%, with no statistically significant differences (*p* > 0.05). Ash content increased markedly in the experimental variants, rising from 2.09% in the control to 6.99% in Variant 4, with statistically significant differences among the formulations (*p* < 0.05).

### 3.4. pH and Water Activity of Sausage Mince Enriched with Protein–Mineral Mass and Protein Hydrolysate

The pH and water activity of sausage mince formulations are presented in Table 4. The control formulation (Variant 1) exhibited a pH value of 6.73. In the experimental variants, pH values ranged from 6.70 to 6.84. Variant 2 showed a pH of 6.79, Variant 3 6.84, and Variant 4 6.70. No statistically significant differences in pH were observed among the formulations (*p* > 0.05). Water activity values ranged from 0.9642 to 0.9719. The control sample showed the highest value (0.9719), while the experimental variants exhibited slightly lower values. The lowest water activity was recorded in Variant 4 (0.9642).

### 3.5. Functional and Technological Properties of Sausage Mince

**Figure 3:**
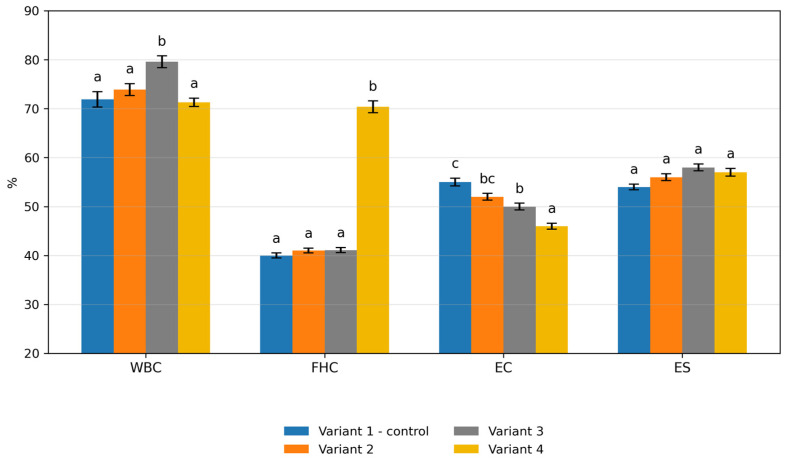
Effect of formulation variants on water-binding capacity (WBC), fat-holding capacity (FHC), emulsifying capacity (EC), and emulsion stability (ES). Different lowercase letters indicate statistically significant differences within each parameter (*p* < 0.05).

The functional and technological properties of sausage mince formulations are presented in Figure 3. The control sample exhibited a water-binding capacity (WBC) of 71.9%. In the experimental variants, WBC values were 73.9% in Variant 2, 79.6% in Variant 3, and 71.3% in Variant 4. Variant 3 showed the highest WBC value and differed significantly from the control and Variant 2 (*p* < 0.05). Fat-holding capacity (FHC) was 40% in the control, 41% in Variant 2, 41.1% in Variant 3, and 70.4% in Variant 4. Variant 4 exhibited a significantly higher FHC compared with the other formulations (*p* < 0.05). Emulsifying capacity (EC) decreased from 55% in the control to 52% in Variant 2, 50% in Variant 3, and 46% in Variant 4, with statistically significant differences observed in Variant 4 (*p* < 0.05). Emulsion stability (ES) values ranged from 54% in the control to 56%, 58%, and 57% in Variants 2–4, respectively. The highest ES value was recorded for Variant 3 (*p* < 0.05 vs. control).

### 3.6. Weight Loss During Heat Treatment of Sausages

**Figure 4:**
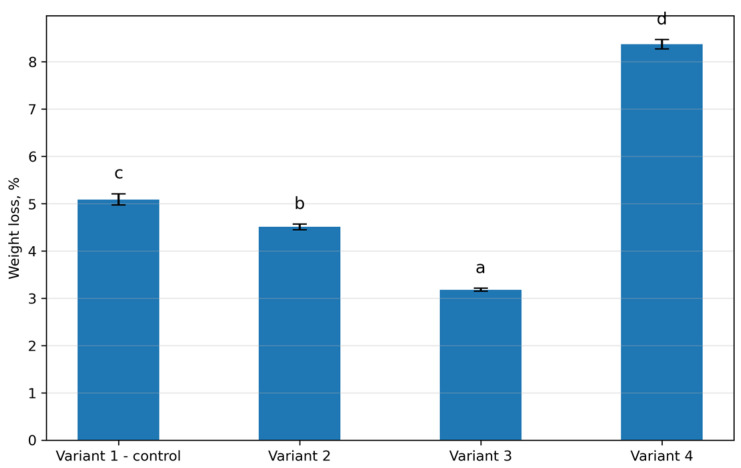
Weight loss of sausages during heat treatment for different formulation variants. Different lowercase letters (a–d) indicate statistically significant differences between variants (*p* < 0.05).

Thermal weight loss of sausage formulations during heat treatment is presented in Figure 4. The control formulation (Variant 1) exhibited a weight loss of 5.09%. In Variant 2, containing 15% protein–mineral mass (PMM) and 2% protein hydrolysate, weight loss decreased to 4.51%, and the difference compared with the control was not statistically significant (*p* > 0.05). The lowest weight loss was observed in Variant 3, where incorporation levels of PMM and protein hydrolysate were 18% and 4%, respectively. In this formulation, weight loss decreased to 3.18%, which differed significantly from both the control and Variant 2 (*p* < 0.05). In contrast, Variant 4, containing the highest levels of PMM (20%) and protein hydrolysate (7%), showed the highest weight loss (8.37%), which was significantly different from all other variants (*p* < 0.05).

### 3.7. Shear Force of Sausages

**Figure 5:**
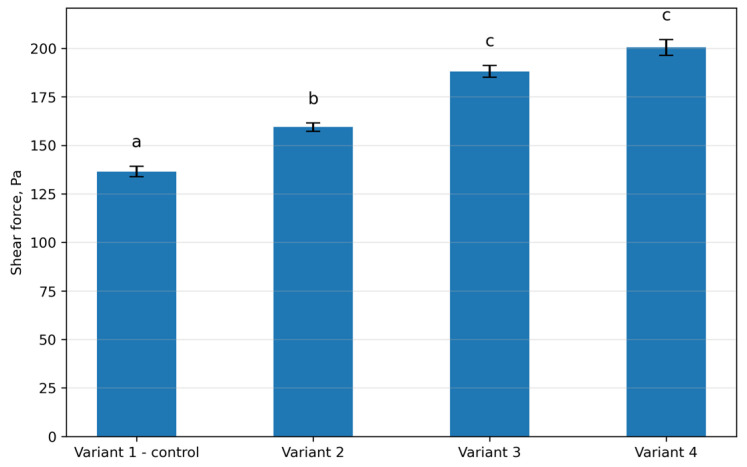
Shear force of sausages with different formulation variants. Different lowercase letters (a–c) indicate statistically significant differences between variants (*p* < 0.05).

Shear force values of sausage samples are presented in Figure 5. The control sample (Variant 1) exhibited a shear force of 136.5 Pa. In Variant 2, containing 15% protein–mineral mass (PMM) and 2% protein hydrolysate, shear force increased to 159.4 Pa, which was significantly higher than that of the control (*p* < 0.05). A further increase in shear force was observed in Variant 3, reaching 188.1 Pa, which differed significantly from both the control and Variant 2 (*p* < 0.05). The highest shear force value was recorded in Variant 4 (200.4 Pa), which was significantly higher than that of the control and Variant 3 (*p* < 0.05).

### 3.8. Color Characteristics of Sausages Enriched with Protein–Mineral Mass and Protein Hydrolysate

Color characteristics of sausage samples are presented in Table 5. The control sample (Variant 1) showed a lightness (L*) value of 70.51 ± 1.28. In Variant 2, L* slightly decreased to 69.38 ± 1.32, while Variant 3 and Variant 4 exhibited lower values of 67.44 ± 0.79 and 66.65 ± 1.45, respectively. However, differences in L* among the variants were not statistically significant (*p* > 0.05).

Redness (a*) increased with increasing levels of PMM and protein hydrolysate. The control sample had an a* value of 7.24 ± 0.10, while Variant 2, Variant 3, and Variant 4 showed higher values of 8.64 ± 0.11, 9.47 ± 0.15, and 9.30 ± 0.16, respectively. These increases were statistically significant (*p* < 0.05). Yellowness (b*) ranged from 11.69 ± 0.15 to 13.59 ± 0.17. Variants 1–3 showed similar b* values, while Variant 4 exhibited the highest value, which differed significantly from the control (*p* < 0.05).

### 3.9. Microstructural Analysis of Sausage Samples

**Figure 6:**
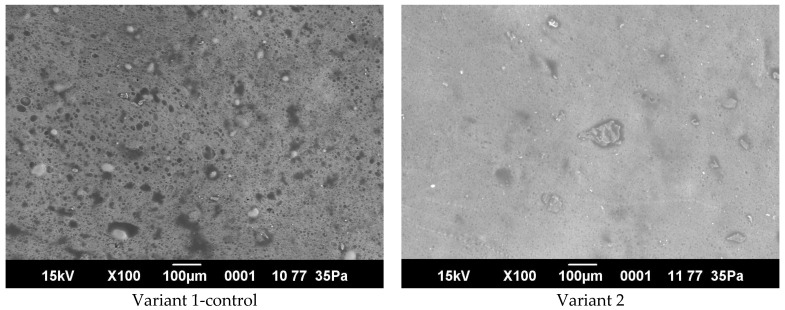

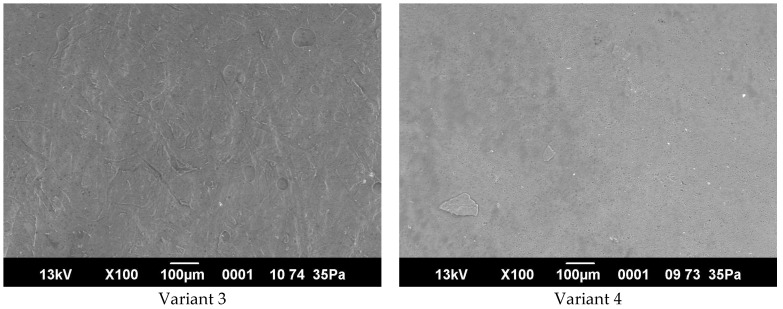
Microstructural characteristics of different variants of sausages.

Microstructural characteristics of sausage samples were examined using scanning electron microscopy (Figure 6). The control sample (Variant 1), formulated only with poultry meat, exhibited a relatively loose and heterogeneous structure. The protein matrix contained visible pores and irregular voids, with unevenly distributed fat inclusions. Variant 2 (15% protein–mineral mass (PMM) and 2% protein hydrolysate) showed a more compact structure compared with the control. The microstructure was characterized by smaller pores and a more uniform distribution of fat droplets within the protein matrix. The most compact and continuous structure was observed in Variant 3 (18% PMM and 4% protein hydrolysate). The micrographs revealed a dense protein matrix with finely dispersed fat inclusions and minimal porosity. In Variant 4 (20% PMM and 7% protein hydrolysate), the matrix appeared dense but less uniform. Local structural irregularities and heterogeneous regions were observed within the protein network.

### 3.10. Organoleptic Characteristics of Sausages Enriched with Protein–Mineral Mass and Protein Hydrolysate

**Figure 7:**
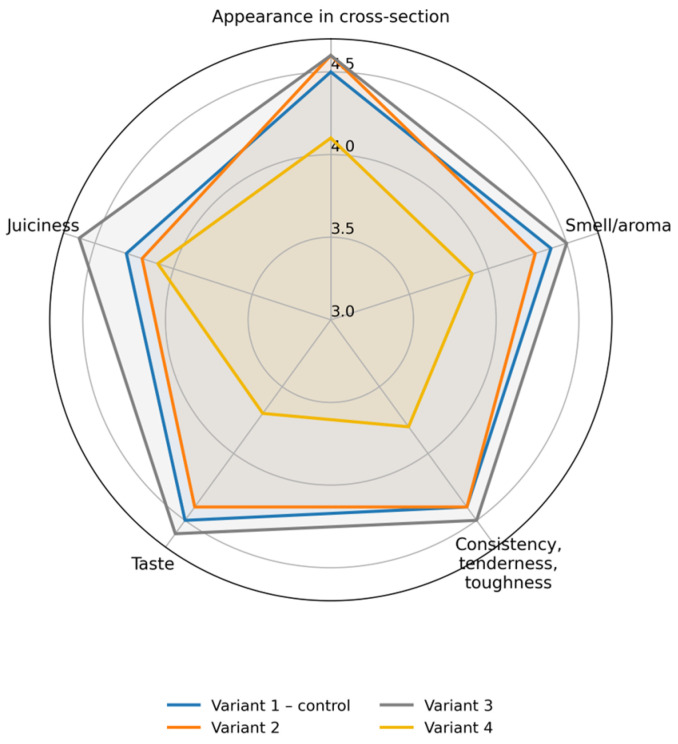
Sensory evaluation scores of different variants of sausages.

**Figure 8:**
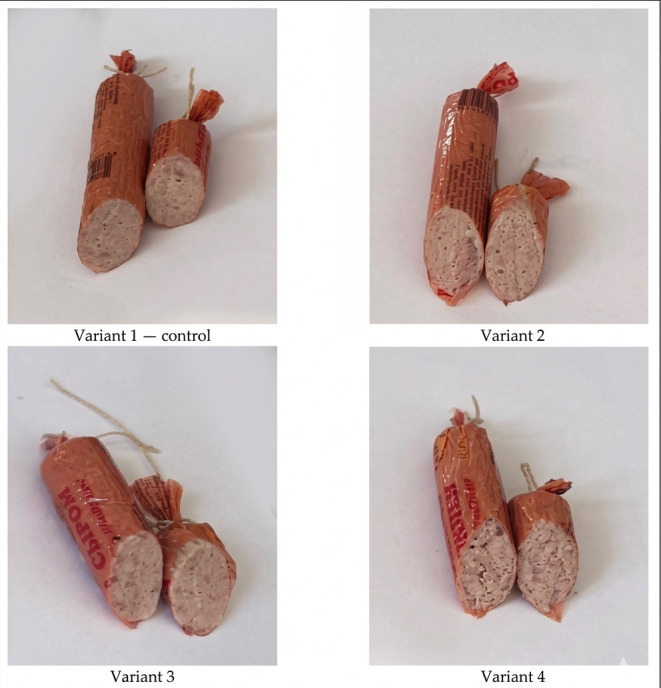
Appearance in cross-section of different variants of sausages.

The sensory profiles of the sausage samples are presented in Figure 7. The control sample (Variant 1) received high scores for appearance in cross-section (4.5), aroma (4.4), texture (4.4), taste (4.5), and juiciness (4.3), with an overall rating of 4.42. Variants 2 and 3 showed comparable sensory characteristics. Appearance scores reached 4.6 in both variants, while aroma scores were 4.3 and 4.5, respectively. Texture scores ranged from 4.4 to 4.5, and taste scores from 4.4 to 4.6. Variant 3 exhibited the highest juiciness (4.6) and the highest overall rating (4.56). These values were not significantly different from those of the control and Variant 2 (*p* > 0.05). In contrast, Variant 4 demonstrated significantly lower sensory scores compared with the other formulations (*p* < 0.05). Appearance, aroma, texture, and taste were rated 4.1, 3.9, 3.8, and 3.7, respectively, while juiciness was 4.1. As a result, Variant 4 obtained the lowest overall rating (3.92). Figure 8 shows the appearance in cross-section of different variants of sausages.

## 4. Discussion

The present results indicate that the technological effect of poultry-derived ingredients depends not only on their chemical composition, but also on how they interact within the sausage matrix. Poultry by-products are increasingly recognized as promising sources of collagen proteins and minerals for the development of functional meat ingredients [29]. However, their practical value in finished products is determined by their ability to improve structure, water retention, and sensory quality without impairing process stability [30]. In this study, chicken bone protein–mineral mass and chicken skin protein hydrolysate modified the sausage system in a formulation-dependent manner, and the best results were obtained when both ingredients were used at intermediate levels.

The starting ingredients showed characteristics expected for collagen-containing materials subjected to enzymatic processing. The protein–mineral mass combined a moderate protein level with a substantial mineral fraction, whereas the hydrolysate was characterized by high protein content, low moisture, low water activity, and strong water- and fat-holding properties. Based on these characteristics, both ingredients can be expected to contribute to nutritional enrichment and matrix stabilization in meat systems. At the same time, the relatively dark color of the protein–mineral mass indicated that its level of incorporation should remain controlled to avoid excessive color changes in the final product.

The compositional changes observed in sausage mince support the proposed mechanism of action. Moisture content decreased progressively, whereas protein and ash contents increased with increasing inclusion of protein–mineral mass and hydrolysate. This effect is attributable to the lower moisture and higher solids content of the added ingredients, especially the freeze-dried hydrolysate, and to the mineral contribution of the bone-derived fraction. Similar trends have been reported in emulsified meat products enriched with collagen-containing materials or bone-derived ingredients [31,32]. Comparable results were reported by Cavalheiro et al., who observed increased protein and ash contents and reduced moisture in mortadella-type sausages when mechanically deboned chicken meat was partially replaced with its protein hydrolysate [33]. In contrast, fat content remained relatively stable, indicating that partial replacement of poultry meat by these ingredients did not destabilize the lipid phase of the emulsion. This is an important distinction from formulations based on mechanically deboned poultry meat, where enrichment is often accompanied by increased fat and reduced protein concentration [31]. Thus, the present approach improved protein and mineral density while preserving formulation balance.

The pH values remained close to neutral in all formulations, indicating that incorporation of these ingredients did not substantially disturb the acid–base balance of the sausage matrix. This is technologically favorable because strong pH shifts can influence protein solubility, hydration, and emulsion formation [34]. Water activity also showed only slight reductions, suggesting somewhat stronger water immobilization in the experimental formulations. These results indicate that the added ingredients were compatible with conventional sausage processing conditions.

The most pronounced effects were observed in the functional and technological properties of the sausage mince. Moderate incorporation of protein–mineral mass and hydrolysate increased water-binding capacity and maintained good emulsion stability, indicating improved hydration and reinforcement of the protein matrix. This behavior is consistent with the presence of collagen-derived proteins and peptides containing numerous hydrophilic groups capable of interacting with water [35]. Similar observations were reported for sausages containing gelatin or gelatin hydrolysates, where low inclusion levels had little effect on basic composition but influenced textural properties such as cohesiveness and chewiness [36]. However, in the present study the technological effect was more pronounced because the hydrolysate was combined with protein–mineral mass, which strengthened the protein network and improved water retention. At the same time, the highest inclusion level produced a marked increase in fat-holding capacity but reduced emulsifying capacity, suggesting that excessive hydrolysate altered the balance between water and fat stabilization. Since collagen-derived peptides generally have lower emulsifying activity than myofibrillar proteins, this result agrees with earlier observations in collagen-enriched emulsified systems [37].

These functional effects were directly reflected in cooking performance. The optimized formulation showed the lowest cooking loss, indicating improved retention of moisture and fat during heating and suggesting the formation of a more cohesive protein network capable of withstanding thermal stress [38]. In contrast, the highest inclusion level produced the greatest cooking loss, indicating that excessive hydrolysate weakened gel continuity and reduced the ability of the matrix to immobilize water. Thus, the action of collagen-derived ingredients was clearly non-linear: moderate incorporation improved performance, whereas excessive incorporation impaired thermal stability.

This interpretation is supported by the mechanical and microstructural results. Shear force increased progressively with increasing levels of protein–mineral mass and hydrolysate, showing that both ingredients reinforced the sausage matrix. A moderate increase in firmness is technologically advantageous because it improves slicing stability and structural integrity. However, excessive shear force may produce an overly dense product with reduced juiciness [39]. The scanning electron micrographs followed the same pattern. The control sample had a looser and more porous structure, whereas intermediate formulations showed a more compact and homogeneous matrix with better dispersed fat inclusions. The most favorable microstructure was observed in Variant 3, which also had the highest water-binding capacity, lowest cooking loss, and balanced shear force. Variant 4 showed excessive compaction and local heterogeneity, supporting the conclusion that too much hydrolysate can disrupt optimal network formation.

These observations also clarify the role of hydrolysate in comparison with previous studies. Pap et al. reported high recovery of soluble collagen peptides from mechanically deboned chicken meat, but also emphasized their limited gelling capacity [40]. This is directly relevant to the present data. The hydrolysate alone would not be expected to form a strong structure, but in combination with bone-derived protein–mineral mass, it appeared to compensate for this limitation and contribute to the formation of a reinforced protein–collagen network. This synergy explains why the best product performance was not achieved at the highest hydrolysate level, but rather at the intermediate level where water-binding capacity reached 79.6%, cooking loss decreased to 3.18%, and shear force reached 188.1 Pa while sensory quality remained high.

Comparison with other hydrolysate studies also supports this interpretation. Liu et al. showed that whey protein hydrolysates improved water retention and reduced structural deterioration in pork patties [41], while Takeda et al. demonstrated functional effects of whey hydrolysates on cured sausage coloration [42]. In the current study, the dominant role of poultry-derived hydrolysate was not antioxidant or color-related, but structural, particularly when combined with the mineral phase. Similarly, collagen hydrolysates combined with bioactive additives have been reported to improve meat quality through antioxidant pathways [43], whereas the present work shows that poultry-derived collagen ingredients can act primarily through physical stabilization of the sausage matrix.

Color and sensory results confirmed the importance of balanced inclusion. Increasing the level of the two functional ingredients reduced lightness and increased redness, likely due to bone-associated pigments, mineral compounds, and thermal reactions during cooking [44]. Moderate increases in redness may improve meat-like appearance, whereas excessive darkening and yellowness at high inclusion levels may reduce consumer appeal. Similar trends were reported by Jin et al., who observed that sausages containing mechanically deboned chicken meat hydrolysates showed increased redness but lower flavor and overall acceptability during storage [45]. Sensory evaluation was consistent with the instrumental data. Moderate incorporation did not impair acceptability and, in the case of Variant 3, resulted in the highest overall sensory score. This formulation combined acceptable appearance, pleasant aroma, improved juiciness, and balanced texture. By contrast, the highest inclusion level led to lower sensory ratings, especially for texture and taste, which corresponded well to the increased firmness and higher cooking loss observed instrumentally.

Taken together, the data show that the main contribution of this study lies not only in producing hydrolysate and protein–mineral ingredients from poultry by-products, but in demonstrating how these ingredients function in a finished meat product. The results show that chicken skin hydrolysate and bone-derived protein–mineral mass can improve composition, process stability, and sensory quality when used at balanced levels. This shifts the practical focus from ingredient recovery alone toward formulation design for direct food application and supports the use of poultry by-products in value-added, more resource-efficient sausage production.

## 5. Conclusions

This study addressed the underutilization of protein- and mineral-rich poultry by-products by evaluating their use as functional ingredients in emulsified sausages. Controlled enzymatic processing of chicken skin and bones yielded a protein hydrolysate and a protein–mineral mass with stable physicochemical and functional properties suitable for meat systems. Their incorporation increased protein and mineral content, improved water-binding capacity and emulsion stability, and reduced cooking losses through the formation of a reinforced protein–collagen network. Sensory and color analyses confirmed that moderate inclusion levels preserved the typical quality of poultry sausages. The combination of 18% protein–mineral mass and 4% protein hydrolysate provided the best balance between yield, texture, and sensory acceptability, whereas higher substitution levels impaired structure and stability. Scientifically, this work defines practical inclusion limits and clarifies structure–function relationships of collagen-derived poultry ingredients in emulsified products. For the industry, the results support cost-effective, sustainable production and reduced reliance on imported additives. Future work should address shelf-life stability and scaling to industrial processing conditions.

## Figures and Tables

**Table 1:** Formulation of sausage mince variants with protein–mineral mass and protein hydrolysate (%).

Ingredient	Variant 1 (Control)	Variant 2	Variant 3	Variant 4
Poultry meat	76.4	60.9	55.9	50.9
Protein–mineral mass	0	15.0	18.0	20.0
Protein hydrolysate	0	2.0	4.0	7.0
Starch	1.5	0.0	0.0	0.0
Curing mixture	1.4	1.4	1.4	1.4
Table salt	0.3	0.3	0.3	0.3
Spice mixture	0.4	0.4	0.4	0.4
Water	20.0	20.0	20.0	20.0

**Table 2:** Physicochemical, functional, rheological, and color characteristics of protein–mineral mass obtained from chicken bones (n = 5).

Indicator	Mean ± SD
Water, %	40.95 ± 0.84
Protein, %	32.61 ± 0.40
Fat, %	7.79 ± 0.07
Ash, %	18.65 ± 0.48
pH	6.93 ± 0.14
Water activity	0.9701 ± 0.020
WBC*, %	45.9 ± 0.92
WHC, %	40.9 ± 0.84
FHC, %	41.1 ± 0.76
EC, %	48.0 ± 1.02
ES, %	18.0 ± 0.28
Viscosity, Pa·s	7.77 ± 0.12
Shear stress, mPa	5556.9 ± 91.7
Yield Stress, kPa	0.612 ± 0.010
Color	
L*	48.11
a*	26.11
b*	11.66

Data are presented as mean ± standard deviation (n = 5 independent samples). WBC*—water-binding capacity; WHC—water-holding capacity; FHC—fat-holding capacity; EC—emulsifying capacity; ES—emulsion stability.

**Table 3:** Physicochemical, functional, and organoleptic characteristics of chicken skin protein hydrolysate.

Indicator	Mean ± SD
Water, %	6.14 ± 0.15
Fat, %	4.82 ± 0.12
Ash, %	36.79 ± 0.99
Protein, %	52.25 ± 1.51
pH	6.19 ± 0.12
Water activity	0.3452 ± 0.006
WHC*, %	142 ± 3
FHC, %	125 ± 2
Organoleptic properties of hydrolysates	
Type, appearance	Fine powder
Color	light beige
Odor	no odor

Data are presented as mean ± standard deviation (n = 5 independent samples). WHC*—water-holding capacity; FHC—fat-holding capacity.

**Table 4:** pH and water activity of sausage mince with different formulation variants.

Variant	pH	Water Activity (a_w_)
Variant 1 (Control)	6.73 ± 0.11 ^a^	0.9719 ± 0.011 ^a^
Variant 2	6.79 ± 0.10 ^a^	0.9674 ± 0.018 ^a^
Variant 3	6.84 ± 0.09 ^a^	0.9692 ± 0.016 ^a^
Variant 4	6.70 ± 0.07 ^a^	0.9642 ± 0.013 ^a^

^a^ Similar lowercase letters indicate no statistically significant differences within the columns (*p* > 0.05).

**Table 5:** Color characteristics (CIE Lab*) of sausages with different formulation variants.

Variant	L* (Lightness)	a* (Redness)	b* (Yellowness)
Variant 1 (Control)	70.51 ± 1.28 ^a^	7.24 ± 0.10 ^a^	12.07 ± 0.22 ^ab^
Variant 2	69.38 ± 1.32 ^a^	8.64 ± 0.11 ^b^	11.69 ± 0.15 ^a^
Variant 3	67.44 ± 0.79 ^a^	9.47 ± 0.15 ^c^	12.44 ± 0.12 ^b^
Variant 4	66.65 ± 1.45 ^a^	9.30 ± 0.16 ^c^	13.59 ± 0.17 ^c^

^a–c^ Different lowercase letters indicate statistically significant differences within the columns (*p* < 0.05).

## Data Availability

The original contributions presented in this study are included in the article/Supplementary Material. Further inquiries can be directed to the corresponding author.

## Supplementary Materials

The following supporting information can be downloaded at: https://www.mdpi.com/article/10.3390/foods15061091/s1, Figure S1: Schematic diagram of the experimental design.
