# Supplementary figures and images for: Effects of Chicken Skin Protein Hydrolysate and Bone Protein–Mineral Mass on the Quality of Emulsified Poultry Sausages
Source: Foods. 2026 Mar 20;15(6):1091. doi: 10.3390/foods15061091 (PMC13025936; doi:10.3390/foods15061091)

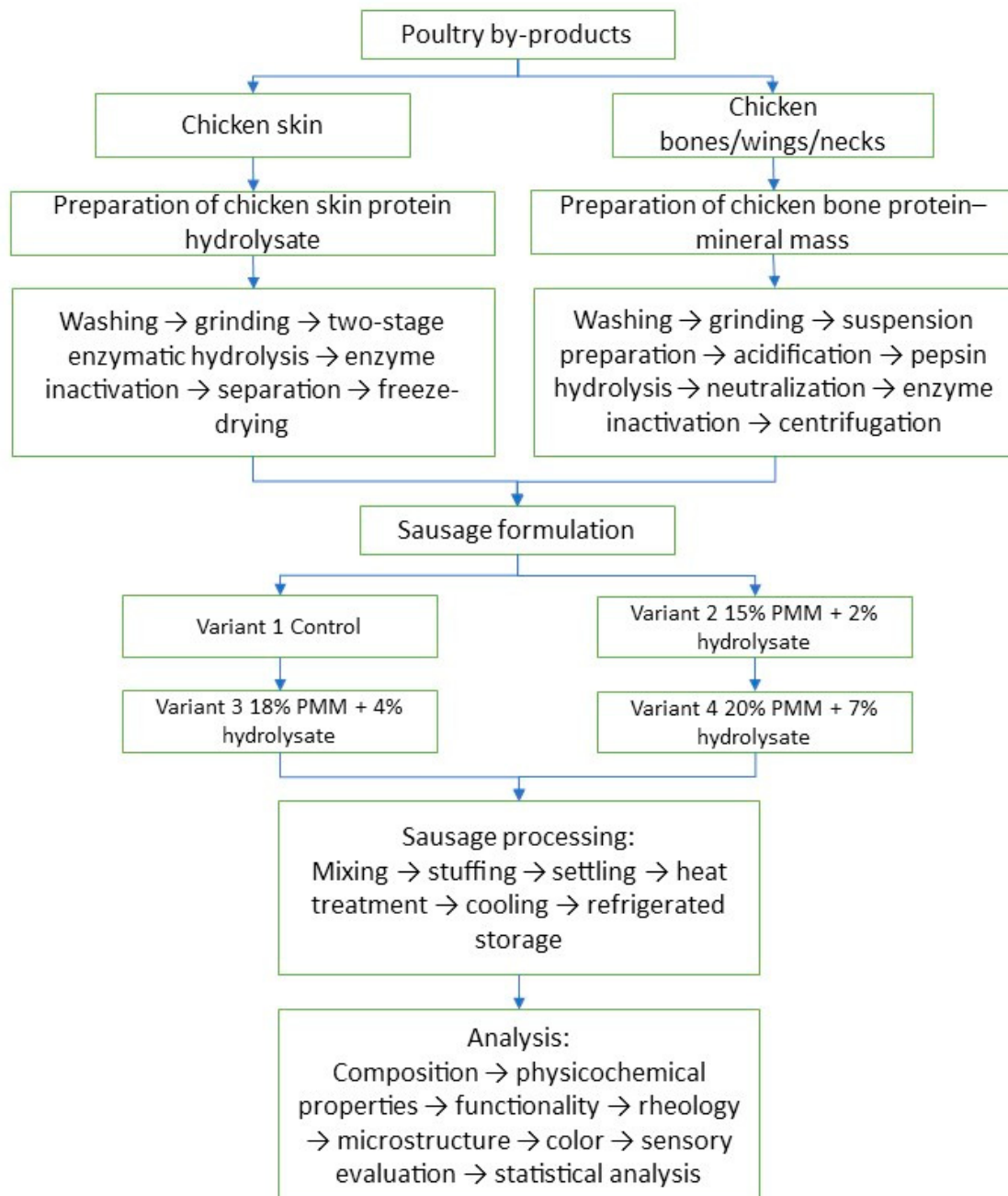

**Figure S1.** Schematic diagram of the experimental design.

Supplement: Supplementary file 1 [file foods-15-01091-s001.zip › foods-4193186-supplementary.pdf]
